# Upregulation of the long noncoding RNA GJA9‐MYCBP and PVT1 is a potential diagnostic biomarker for acute lymphoblastic leukemia

**DOI:** 10.1002/cnr2.2115

**Published:** 2024-07-12

**Authors:** Kamal Shahamiri, Arash Alghasi, Najmaldin Saki, Hossein Teimori, Gholam Abbas Kaydani, Setare sheikhi

**Affiliations:** ^1^ Cellular and Molecular Research Center, Basic Health Sciences Institute Shahrekord University of Medical Sciences Shahrekord Iran; ^2^ Thalassemia & Hemoglobinopathy Research center, Health research institute Ahvaz Jundishapur University of Medical Sciences Ahvaz Iran; ^3^ Department of Laboratory Sciences, School of Allied Medical Sciences Ahvaz Jundishapur University of Medical Sciences Ahvaz Iran; ^4^ Department of Hematology and Blood Transfusion, School of Allied Medical Sciences Tehran University of Medical science Tehran Iran

**Keywords:** acute lymphoblastic leukemia, LncRNA GJA9‐MYCBP, PVT1

## Abstract

**Background:**

Acute lymphoblastic leukemia (ALL) is the most common type of blood cancer in children. Aberrant expression of long noncoding RNAs (lncRNAs) may set stages for ALL development. LncRNAs are emerging as a novel diagnostic and prognostic biomarker for ALL. Herein, we aimed to evaluate the expression of lncRNA GJA9‐MYCBP and PVT1 in blood samples of ALL and healthy individuals.

**Methods:**

As a case–control study, 40 pairs of ALL and healthy individual samples were used. The expression of *MYC* and each candidate lncRNA was measured using quantitative real‐time PCR. Any possible association between the expression of putative noncoding RNAs and clinicopathological characteristics was also evaluated.

**Results:**

LncRNA GJA9‐MYCBP and PVT1 were significantly upregulated in ALL samples compared with healthy ones. Similarly, mRNA levels of MYC were increased in ALL samples than control ones. Receiver operating characteristic curve analysis indicated a satisfactory diagnostic efficacy (*p*‐value <.0001), suggesting that lncRNA GJA9‐MYCBP and PVT1 may serve as a diagnostic biomarker for ALL. Linear regression analysis unveiled positive correlations between the expression level of MYC and lncRNA GJA9‐MYCBP and PVT1 in ALL patients (*p*‐values <.01).

**Conclusions:**

In this study, we provided approval for the clinical diagnostic significance of lncRNA GJA9‐MYCBP and PVT1that their upregulations may be a diagnostic biomarker for ALL.

## 1 INTRODUCTION

Malignant transformation of either myeloid or lymphoid hematopoietic progenitors in bone marrow causes acute leukemias.^1^ The leukemic cells often overcome some regulatory barriers and evade controlling terminal differentiation.^2^ Among different types of leukemias, acute lymphoblastic leukemia (ALL) is by far the most common type of blood cancer in children and is responsible for more than 75% of all childhood leukemias.^3^ Besides, more than 80% of childhood ALL are categorized into B‐cell type.^4^

Despite certain diagnostic and treatment advancements, the survival rate of ALL patients remains low.^5^ Hence, in addition to casting light on underlying mechanisms of ALL progression, the identification of novel diagnostic biomarkers seems urgent for better disease management.^6^ In this context, long noncoding RNAs (lncRNAs) have been introduced as a novel class of molecules^7^, ^8^, ^9^, ^10^ that may have roles in ALL initiation, progression, and development. In fact, lncRNAs are a class of noncoding RNAs (with about 200 nucleotides in length) that can modulate target gene expression at multiple levels in pathophysiological conditions.^11^, ^12^, ^13^ LncRNAs are of paramount importance for a broad range of cellular functions such as chromatin organization, mRNA stability, and the regulation of translation.^11^, ^12^, ^13^ In essence, lncRNAs contribute to various biological processes that are important in cancer biology such as the regulation of cell proliferation,^14^ differentiation,^11^ angiogenesis, apoptosis, and cell motility.^11^ The aberrant expression of lncRNAs has been documented in different human diseases, particularly in cancer.^10^

LncRNAs may function during carcinogenesis as potential tumor suppressors or oncogenes.^7^ Some scrapes of evidence have shown that dysregulation of lncRNAs may set stages toward leukemia development (reviewed in Reference 15). Because they are easily detected in biofluids and exhibit a consistent expression during cellular differentiation, lncRNAs may serve as a diagnostic and prognostic biomarker in different cancers.^10^

Both *N‐Myc* and *C‐Myc* are expressed during the maturation and expansion of the earliest B‐cell precursors, however, the expression of *L‐Myc* has not been documented in any stem/progenitor cells (reviewed in Reference 16). After B‐cell activation, only *C‐Myc* is expressed in mature B cells.^17^ C‐Myc upregulation has been detected in human ALL via various mechanisms, for example, translocations t(8;14), t(8;22), and t(2;8), aberrant C‐Myc stability, prolonged C‐Myc protein half‐life, and activating mutations in NOTCH1.^16^ High expression of MYC and C‐Myc targets have been recently identified in T‐ALL (reviewed in Reference^7^). In fact, MYC can be regulated by different lncRNAs, both at transcriptional and posttranscriptional levels.^18^ MYC also can modulate the expression of a bunch of candidate lncRNAs that many of which can influence cancer cell viability and proliferation.^18^, ^19^ In other words, this protein binds to the promoter of different lncRNAs and controls their expression.^20^ This may elaborate on the pro‐oncogenic roles for MYC.

Recent studies have also indicated that MYC binding protein (MYCBP) and its related lncRNAs are associated with tumorigenesis in different cancers such as colon cancer and glioma.^21^, ^22^ LncRNA GJA9‐MYCBP, also known as lnc‐GJA9‐1, has been identified to target hsa‐miR‐7a‐5p, a molecule that may play important role in leukemia.^23^ However, the potential role of this lncRNA in ALL, to the best of our knowledge, is still unclear. As another important lncRNA, plasmacytoma variant translocation 1 (PVT1) is located downstream of MYC on 8q24.^24^ This lncRNA plays important role in both normal and malignant conditions.^24^ The expression of lncRNA PVT1 is linked to increased proliferation and invasion of osteosarcoma,^25^ gastric cancer,^26^ non‐small cell lung cancer,^27^ cervical cancer,^28^ and melanoma.^29^ It has been suggested that the upregulation of lncRNA PVT1 may result in low overall survival rates in different cancers, indicating its diagnostic and prognostic roles.^27^, ^30^ Using different intracellular pathways, PVT1 potentiates the tumorigenic activity of leukemic cells.^31^ According to Jin et al., lncRNA PVT1 interacts with MYC and its downstream molecules to promote tumorigenesis in a synergistic manner^32^; for instance, in ALL, circulating PVT1 upregulates the expression of C‐Myc and anti‐apoptotic BCL‐2 proteins.^31^

Herein, using literature review and after nominating some important MYC‐associated lncRNAs. We selected two important lncRNAs. We aimed to assess whether lncRNA GJA9‐MYCBP and PVT1 may contribute to ALL pathogenicity and also to show whether their expressions are associated with some clinicopathological features. We also evaluated whether the putative lncRNAs may be used as an ALL diagnostic biomarker. The results of this study can highlight the importance of lncRNA GJA9‐MYCBP and PVT1 as ALL diagnostic biomarkers.

## 2 MATERIALS AND METHODS

### 2.1 Study population

The study protocol was approved by the Ethics Committee of Shahrekord University of Medical Sciences, Shahrekord, Iran, under the declaration code of “IR.SKUMS.REC.1399.273.” Written informed consent was signed by the legal guardians of each participant under the Declaration of Helsinki.

We recruited 40 ALL patients (26 males and 14 females), aged 1–16, who were admitted to the Department of Cellular and Molecular Research Center, Shahrekord University of Medical Sciences, Shahrekord, Iran, from 2020 to 2021. In detail, 16 individuals were 1–5, 11 people were 6–10, and 13 people were 11–16 years old. To diagnose, a bone marrow aspiration was carried out according to the World Health Organization (WHO) criteria based on disease characteristics including immunophenotype, blasts morphology, and chromosomal, and molecular abnormalities.^33^ Newly diagnosed ALL patients were treated based on Berlin‐Frankfurt‐Munster (BFM) protocol.^34^

Moreover, a total of 40 control individuals embracing 21 males, and 19 females were included who were negative for any malignancies. In detail, 17 individuals were 1–5, 15 from 6 to 10, and 8 people were 11–16 years old. We excluded the individuals with a positive history of malignancies and acute or chronic inflammatory diseases. All clinical and demographical features of each participant and inclusion/exclusion critria were put forth in Table 1, 2.

**TABLE 1:** The important clinicopathological features in ALL patients.

Characteristics		No. of patients (*n* = 40)	(%)
Sex	Male	26	65%
	Female	14	35%
Age (year)	1–5	16	40%
	6–10	11	27.5%
	11–16	13	32.5%
MRD of 29 days	≥1%	6	15%
	<1%	34	85%

Abbreviation: MRD, measurable residual disease.

**TABLE 2:** The criteria for inclusion/exclusion the study.

Inclusion criteria	Exclusion criteria
Approved ALL with WHO criteria	Positive history of malignancies
Age 1–16	Positive history acute or chronic inflammatory diseases
Treated with chemotherapy	

Abbreviation: WHO, World Health Organization.

### 2.2 Functional enrichment analysis

To elaborate regulatory functions of each candidate lncRNA and also their possible targets, lncRNA2Target v.2.0^35^ was used. Just in case, the LncSEA tool was used to explore the target genes, transcription factors, and RNA binding proteins.^36^ LncBase Predicted/Experimental v.2^37^ was also used to predict any potential interactions between the candidate lncRNAs and MYC, particularly in bone marrow tissues. The threshold was set as a score >0.7. To identify any probable interaction between the lncRNAs and other proteins, and also to show the important candidate pathways, we used SFPEL‐LPI^38^ and focused on the “Top 5” interactions. RNA Interactome Database (RNAInter)^39^ was employed in order to elaborate any probable RNA–RNA interactions.

### 2.3 RNA and lncRNA extraction

A total of 10 mL of blood sample was obtained from each participant and placed in an ethylenediaminetetraacetic acid‐containing tube. Total RNA was isolated from blood samples of ALL patients and healthy individuals using Trizol Reagent (Thermo Fischer Scientific, Ma, USA) according to the manufacturer's instruction. In order to eliminate any probable DNA contamination, RNA samples were treated with RNase‐free DNase (Qiagen, Valencia, CA, USA). After extraction, the quality of RNA samples was evaluated using Qubit 4 Fluorometer (Thermo Fischer Scientific) and further analyzed by 2% agarose gel electrophoresis.

In order to extract lncRNAs, about 2.0 μg of each RNA sample was reverse‐transcribed into complementary DNA (cDNA) using RevertAid First Strand cDNA Synthesis Kit (GENEALL, Korea) in a total of 20.0 μL reaction mixture. This kit uses a random hexamer and oligo(dT)_18_ primers that are necessary for lncRNA replication.

### 2.4 quantitative real‐time PCR

Quantitative real‐time PCR (RT‐qPCR) was performed on an ABI StepOne Sequence Detection System (Applied Biosystems, VIC, Australia) using SYBR® Premix Taq™II master mix (Berlin, Germany) tally with the manufacturer's instructions. The expression of the *MYC* and the candidate lncRNAs (i.e., GJA9‐MYCBP and PVT1) was measured according to the comparative threshold cycle (Ct) method relative to the *Beta‐actin* (*ACTB*), as a housekeeping gene. The calculations were based on 2^−ΔCt^ where ΔCt = Ct (Target)^−Ct^ (Reference).^40^ We also calculated fold changes according to the 2^−ΔΔCt^ method.^40^ The following forward (F) and reverse (R) primers were used: *ACTB*‐F, 5′‐TGGGCATCCACGAAACTAC‐3′, *ACTB*‐R: 5′‐GATCTCCTTCTGCATCCTGT‐3′; *MYC*‐F: 5′‐GTTGGGAGGAAGGTGAGGAA‐3′, and *MYC*‐R: 5′‐CCTCTGGGGTTTGCGAGATA‐3′; lncRNA *GJA9‐MYCBP*‐F: 5′‐TTGTACGGGTTCCCATGAAT‐3′, and lncRNA *GJA9‐MYCBP*‐R: 5′‐AACAGCACAGAAAGGCCAGT‐3′; lncRNA *PVT1*‐F: 5′‐GATTCACAAGCCCCACCAAG‐3′, and lncRNA *PVT1*‐R: 5′‐CGTTTTCCCACAGTGATGCT‐3′. Melting curves were used to analyze the specificity of PCR products at 95°C for 15 s, 60°C for 30 s, and 90°C for 15 s.

### 2.5 Statistical analysis

All data are present according to the mean ± standard deviation (SD) of at least three independent experiments. To do statistical analysis, SPSS version 27.0 (SPSS Inc., Chicago, IL, USA) and GraphPad Prism v.8.0 (GraphPad, San Diego, USA) were employed. The statistical analysis was performed based on the Student's *t*‐test and Mann–Whitney test and we considered *p*‐values <.05 statistically significant. The receiver operating characteristic (ROC) curves were used to show the diagnostic value of the signature, especially by calculating the area under the curve (AUC) with at least 95% of confidence intervals (CI). Any correlation between the expression of *MYC* and each candidate lncRNA was performed using the Spearman correlation coefficient.

## 3 RESULTS

### 3.1 Basic characteristics

The anthropometric characteristics were recorded for each participant (Table 1) before and during the examination. There were 26 males and 14 females in the patient group, and 21 males and 19 females in the control group. The mean age of the patient and control individuals was 5.6 and 5.8 years, respectively There were 22 patients B‐All and 8 patients with T‐All. Interestingly, no significant differences were observed between these two groups in terms of their age and sex.

### 3.2 Functional enrichment analysis

To show the regulatory functions of each candidate lncRNA and their possible gene targets, lncRNA2Target v.2.0 was used. The findings showed that lncRNA PVT1 contributes to the progression or development of acute promyelocytic leukemia. Besides, using this database, we also determined that lncRNA GJA9‐MYCBP may be involved in normal tissue homeostasis; however, further information, for example, regarding its roles in cancer biology, was shrouded in mystery.

**FIGURE 1:**
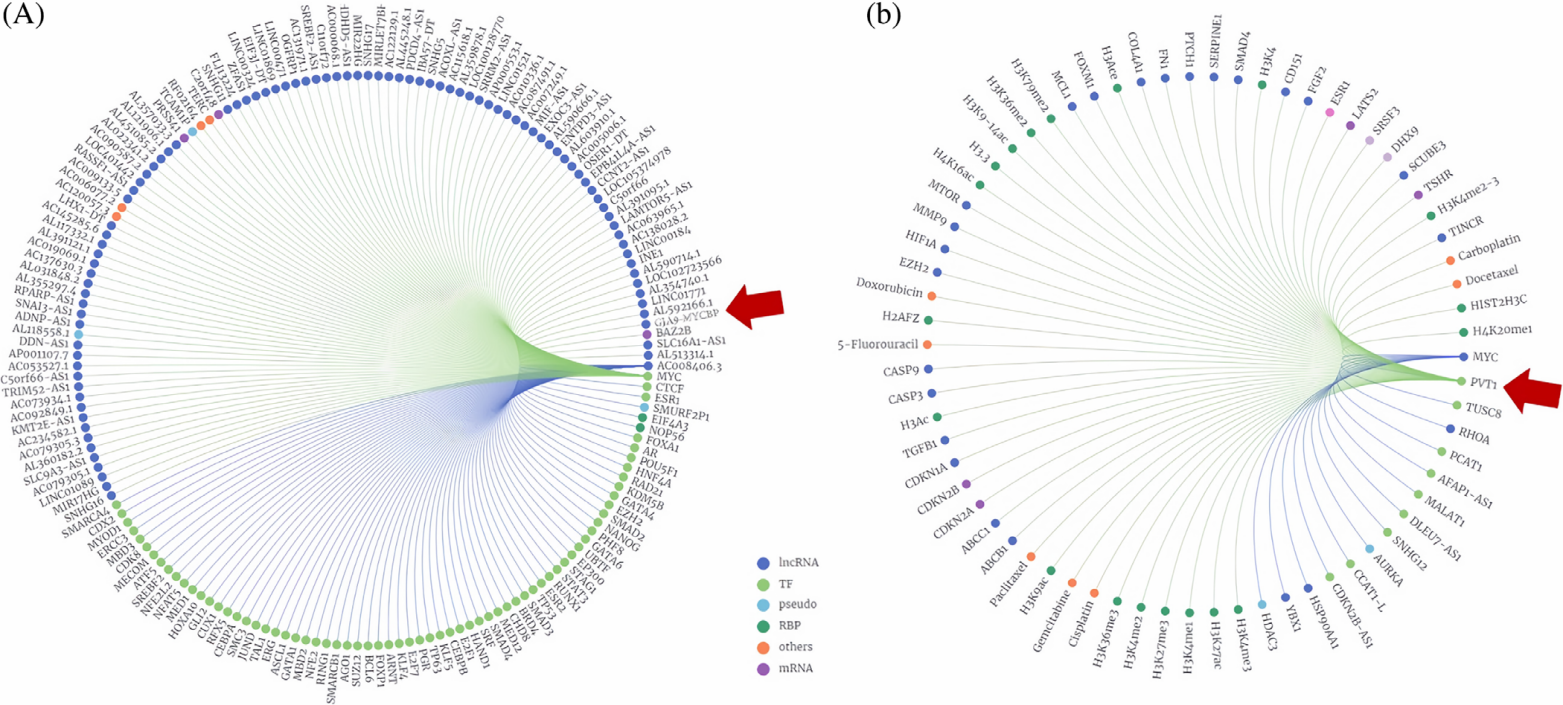
Target prediction for each candidate lncRNA. (A) GJA9‐MYCBP was detected to target MYC (red arrow shows this lncRNA). (B) MYC was also identified as a direct target of PVT1. The graphs were depicted using RNAInter (https://www.rna‐society.org/rnainter). lncRNAs, long noncoding RNAs; PVT1, plasmacytoma variant translocation 1.

**FIGURE 2:**
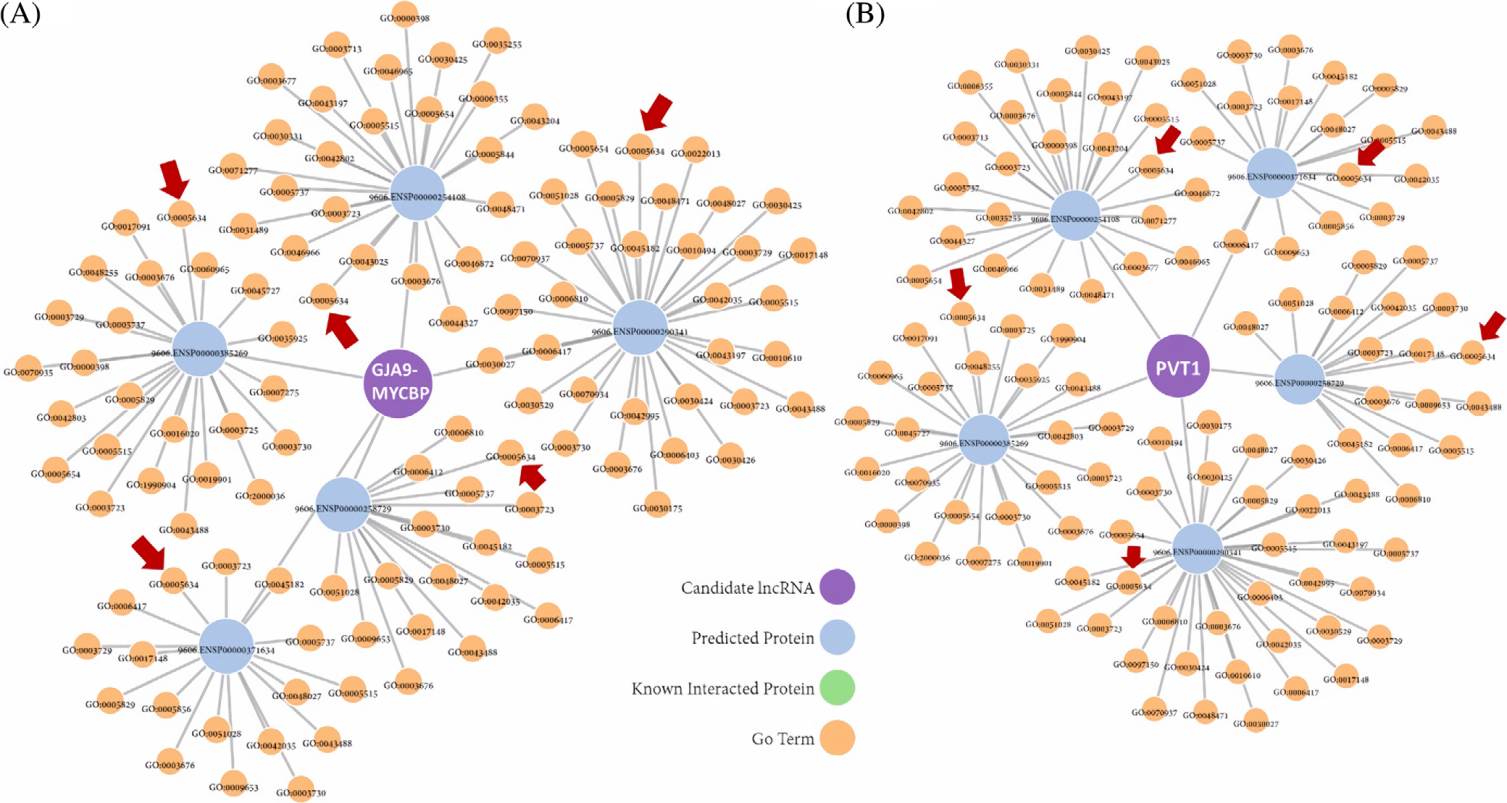
Visualization of “Top 5” predicted interacting molecules for the lncRNA GJA9‐MYCBP and PVT1, (A) GJA9‐MYCBP, and (B) PVT1. The purple node shows the candidate lncRNAs, while the blue circles highlight the predicted interacting proteins. The corresponding GO terms (Orange nodes) of each interacting protein were depicted based on the Quick GO database (https://www.ebi.ac.uk/QuickGO/). MYC protein was shown using a red arrow. lncRNAs, long noncoding RNAs; PVT1, plasmacytoma variant translocation 1.

Regarding lncRNA PVT1, we predicted that it may function through MYC protein. Thence, any possible RNA–RNA interactions were determined using the RNAInter. Using this tool, we interestingly showed that MYC is predicted as a direct target for lncRNA PVT1 and GJA9‐MYCBP, respectively (Figure 1A,B). LncSEA and SFPEL‐LPI were also utilized to verify these kinds of results. In predicted mode, they showed that these lncRNAs may function through MYC or MYC‐downstream molecules or they target these kinds of molecules (Figure 2A,B; Tables S1 and S2).

### 3.3 *MYC* expression analysis in B‐ALL and T‐ALL samples

Five‐fold serial dilutions of cDNA samples were used to measure the reaction efficiency of each primer set. The amplification efficiency for *MYC*, the candidate lncRNAs, and *ACTB* was nearly equal, confirmed by a linear correlation, showing that the assay is accurate for relative expression quantification. Moreover, dissociation curve analysis verified the specificity and uniqueness of each product. In other words, a single and sharp curve was obtained in each sample, showing that neither primer‐dimer nor nonspecific products were formed during reactions (Figure S1).

**FIGURE 3:**
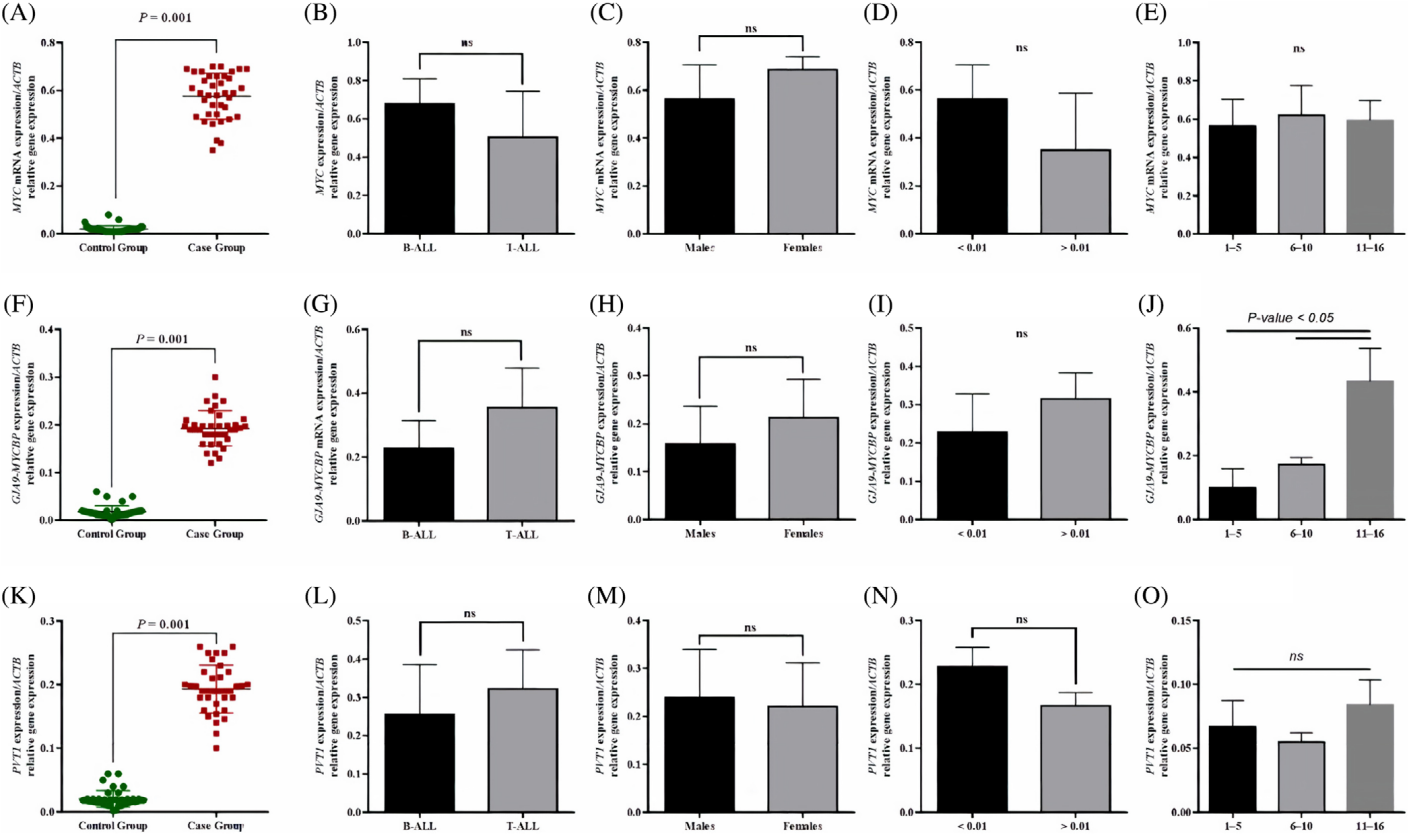
The expression analysis of MYC, GJA9‐MYCBP, and PVT1 in ALL (*n* = 40) and healthy individuals (*n* = 40). (A) The expression of MYC was increased in blood samples derived from the ALL patients than the control group (*p*‐value = 0.001). No differences were detected in terms of ALL subtypes (B), sex (C), multi‐drug resistance (MDR) status (D), and age (E). F) The expression analysis using RT‐qPCR showed the upregulation of GJA9‐MYCBP in ALL samples (*P*‐value = .001). ΔCt values for each sample were calculated by normalization using *ACTB* as an internal control. No differences were detected in terms of ALL subtypes (G), sex (H), and MDR status (I). (J) The expression of GJA9‐MYCBP was increased in ALL patients who were 11–16 years old. (K) mRNA levels of PVT1 were significantly increased in ALL patients compared to the control group (*p*‐value = .001). No significant differences were detected in terms of ALL subtypes (L), sex (M), MDR status (N), and age (O). ALL, acute lymphoblastic leukemia; PVT1, plasmacytoma variant translocation 1; RT‐qPCR, quantitative real‐time polymerase chain reaction (PCR).

We evaluated the expression of *MYC* in 40 pairs of ALL and control blood samples. We showed that the *MYC* expression was greater in patients' samples than those of control ones (*p‐*value <.001; Figure 3A). We also measured the expression levels of *MYC* in B‐ALL and T‐ALL subtypes, however, the results showed no significant difference between the two groups (Figure 3B). We also investigated whether the expression of *MYC* is dependent on patients' sex and measurable residual disease (MRD) status or not. The persistence of MRD after intensive chemotherapy is a major prognostic factor in adult patients with acute leukemia.^41^ Our results showed no significant differences in terms of these variables (Figure 3C,D). We also showed no significant differences among the three groups of patients in terms of their ages (Figure 3E).

### 3.4 *GJA9‐MYCBP* and *PVT1* expression in ALL samples

Upregulation of lncRNA *GJA9‐MYCBP* was identified in ALL samples compared with control ones (*p*‐value <.001; Figure 3F). This upregulation, however, did not show any significant differences in terms of patients' ALL subtypes, MDR types, and sex. On the other hand, the expression of this lncRNA was significantly increased in the age group of 11–16 years compared with other age groups (Figure 3G–J).

The results of RT‐qPCR also showed that lncRNA *PVT1* expression was significantly increased in patients' samples when they were compared to the control group. There were no significant differences between the two groups in terms of patients' ALL subtypes, sex, MDR, and age (Figure 3K–O).

### 3.5 Correlation between the expression levels of *MYC* and candidate lncRNAs

**FIGURE 4:**
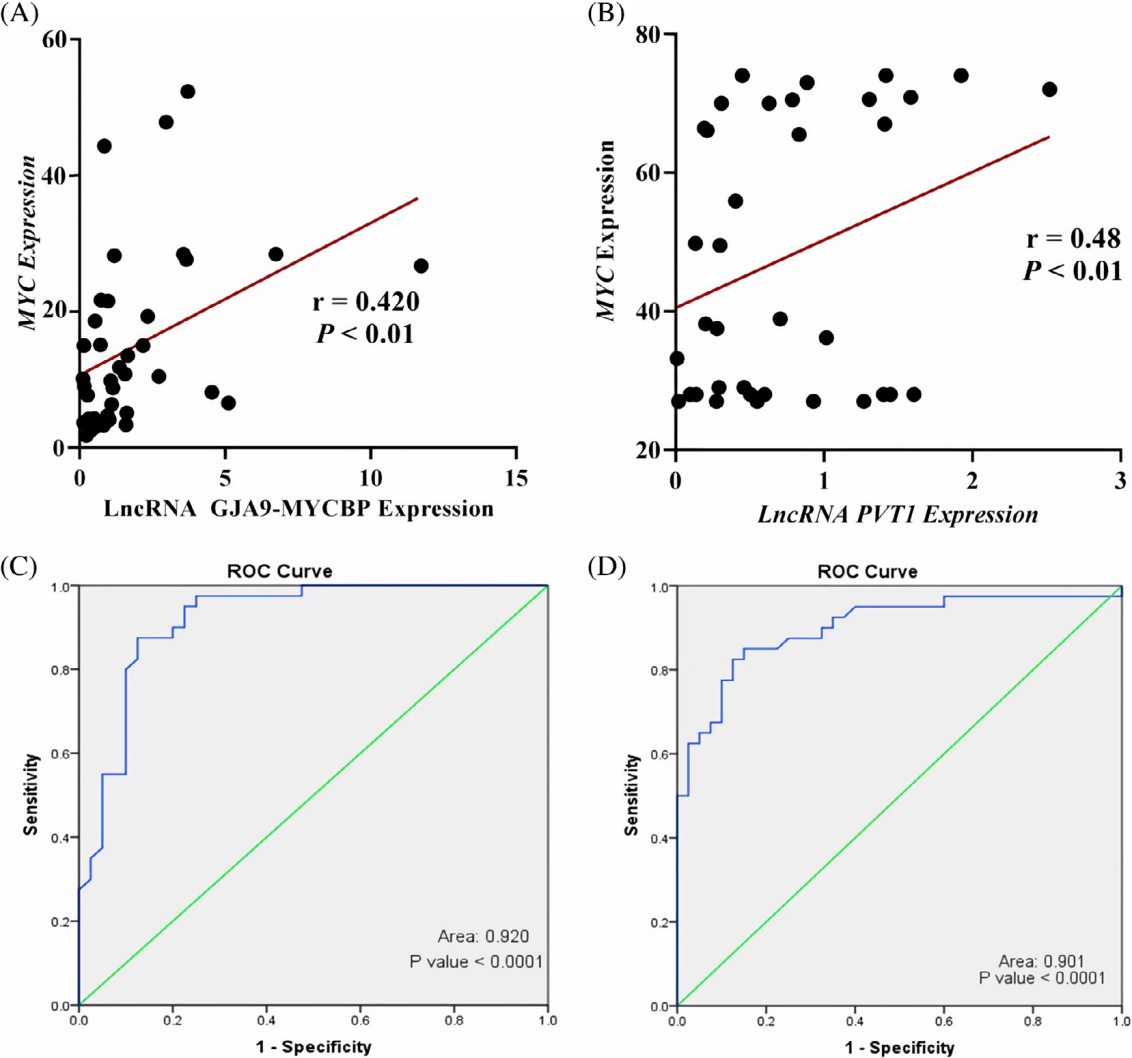
(A)) A positive correlation was detected between the expression of *MYC* and *GJA9‐MYCBP*. (B) The expression analysis of lncRNA PVT1 and MYC revealed statistically a positive correlation. (C) ROC curve analysis of *GJA9‐MYCBP* showed this lncRNA can discriminate two definite groups with a promising calculated AUC (0.920) and *p*‐value = .0001 (95% CI). (D) PVT1 can be considered a biomarker (AUC = 0.901, *p*‐value = .0001, 95% CI). AUC, area under the curve; CI, confidence intervals; lncRNAs, long noncoding RNAs; PVT1, plasmacytoma variant translocation 1; ROC, receiver operating characteristic.

To show whether there is any possible correlation between the expression of each candidate lncRNA with *MYC* expression, a linear regression analysis was performed. Our results showed a positive correlation between the expression level of GJA9‐MYCBP and the MYC mRNA levels in ALL patients (*r* = .42, *p*‐value <.01; Figure 4A). We also indicated that the expression level of lncRNA PVT1 and MYC were in a positive correlation in ALL patients (*r* = .48, *p*‐value <.01; Figure 4B).

### 3.6 Diagnostic performance of lncRNAs

ROC‐AUC analysis was used to show the sensitivity and specificity of GJA9‐MYCBP and PVT1 as a diagnostic biomarker for ALL. The AUC for these lncRNAs was calculated 0.920 (*p*‐value = .0001) and 0.901 (*p*‐value = .0001), respectively (Figure 4C,D). The data show that these lncRNAs can accurately discriminate ALL patients from healthy individuals, so they can be considered ALL diagnostic biomarkers.

The study underscores the importance of addressing a crucial gap in the field of biomarker‐based detection and diagnosis of ALL. It raises pertinent questions about how the identified lncRNAs, GJA9‐MYCBP, and PVT1, offer advancements over existing biomarkers. Specifically, the inquiry into whether these lncRNAs are present in ALL specimens where traditional biomarkers may not be detected is pivotal. This line of questioning is significant as it delves into the potential uniqueness and complementary value of the identified lncRNAs. If GJA9‐MYCBP and PVT1 demonstrate presence in cases where established biomarkers fall short, it suggests a distinctive and possibly more sensitive role for these lncRNAs in detecting ALL. This exploration is fundamental for understanding the practical implications of incorporating these lncRNAs into diagnostic strategies and how they might improve the precision and reliability of ALL detection compared to existing methods.

## 4 DISCUSSION

ALL is one of the leading causes of cancer‐related death in children worldwide.^42^ Albeit the latest advancements in clinical therapies that led to improving clinical conditions (reviewed in Reference 43), ALL still has a low rate of overall survival.^43^ Therefore, as an effective strategy, making an early and precise diagnosis is of paramount importance.

Among different diagnostic methods, lncRNAs can be used as a biomarker. They have different roles (as oncogenes or tumor suppressors) in tumorigenic development in a variety of cancers, making them an ideal biomarker.^10^, ^13^ Specifically, a substantial number of studies have shown that specific lncRNAs may contribute to leukemia and can be used as diagnostic and/or prognostic biomarkers in such patients.^44^, ^45^ Furthermore, these kinds of molecules are detectable in biofluids of cancer patients, so they can be used to reflect the patients' overall status and used for cancer diagnosis and therapeutic monitoring.^45^ It has been identified that lncRNA GJA9‐MYCBP and PVT1 are aberrantly expressed in different human cancers,^46^, ^47^, ^48^ but it is not clear how these lncRNAs contribute to ALL or serve as a diagnostic biomarker in this kind of cancer. To fill this yawning gap, we investigated the expression of these lncRNAs along with their potential target, *MYC*, in ALL samples.

Upregulation of MYC has been reported in human ALL. In line with this, our results showed that in 40 ALL samples, this gene was upregulated. We also showed that this alternation in gene expression was not associated with ALL subtypes, patients' sex and age, and MRD status. Many studies have investigated if MYC changes lncRNA expression, and they found that not only MYC can exert its pro‐oncogenic roles through promoting the expression of different lncRNAs, but it can be regulated by a variety of lncRNAs. In this study, we showed that there is a positive correlation between the expression of *MYC* and lncRNA GJA9‐MYCBP and PVT1. It is pretty fair to suggest that there is a biological relationship between these lncRNAs and MYC, however, further studies are needed to arrive at a conclusive conclusion.

An accumulating evidence has verified that the aberrant expression of MYCBP‐related lncRNAs may play important roles during cancer development.^21^, ^49^ MYCBP binds to MYC proto‐oncogenes, so, for example, enhances the ability of c‐MYC protein‐promoted tumorigenesis.^50^ The upregulation of MYCBP promoted the invasion and migration of gastric cancer cells.^51^ As an important lncRNA, MYCBP2‐AS1 is associated with improved survival time and chemo‐response in high‐grade serous ovarian cancer.^52^ Despite some research on how MYCBP‐related lncRNAs may contribute to human diseases, the exact roles of GJA9‐MYCBP in the pathophysiology of leukemia are still blanketed in mystery. Herein, we showed that in ALL samples, GJA9‐MYCBP was upregulated in patients' samples compared to the healthy ones. This may highlight some oncogenic roles to this lncRNA. Interestingly, lncRNA GJA9‐MYCBP was significantly upregulated in 11–16 years old patients, so it seems fair to propose that this lncRNA may reflect high‐advanced grades of ALL. We also calculated ROC‐AUC, which indicated that the lncRNA can distinguish ALL and normal samples. We also showed that the expression of GJA9‐MYCBP is not associated with ALL subtypes, MRD status, and sex.

*PVT1* has been identified to co‐amplify with MYC in different tumors.^24^, ^53^ The upregulation of PVT1 contributes to the pathophysiology of different cancers to touch upon ovarian, breast, prostate, mesothelioma, and acute promyelocytic leukemia.^24^ PVT1 increases MYC stability by protecting this protein from phosphorylation and so halters its proteolytic degradation.^32^ A similar mechanism was also documented in acute promyelocytic leukemia cells upon PVT1 knockdown.^54^ PVT1 plays as a double‐edged sword: while the oncogenic ability of MYC is strongly imputed to increased expression of PVT1, this lncRNA may exert some anti‐apoptotic activities.^55^ Besides, PVT1 may contribute to tumor development independently of MYC.^24^ It has been suggested that at high transcription levels, PVT1 may show some oncogenic properties. In this study, we showed that PVT1 was upregulated in ALL samples compared with the normal ones. This upregulation was independent of ALL subtypes, patients' age and sex, and MRD status. ROC‐AUC analysis showed that PVT1 may discriminate ALL and healthy samples with adequate sensitivity and specificity, so it can be considered as a novel diagnostic biomarker for ALL.

One of the most important advantages of using GJA9‐MYCBP and PVT1 as diagnostic markers compared to other existing markers is that, firstly, peripheral blood instead of bone marrow can be used to check them, and they can also be detected at a younger age. A critical examination of whether the identified lncRNAs, GJA9‐MYCBP, and PVT1, exhibit synergistic or additive effects when combined with other biomarkers, thereby enhancing the overall efficacy of disease diagnosis. This consideration is crucial for understanding the potential value of incorporating these lncRNAs into a multi‐marker approach for ALL detection. The concept of synergy or additive effects suggests that the combination of these lncRNAs with established biomarkers could lead to improved diagnostic accuracy. If the lncRNAs contribute uniquely or enhance the performance of existing markers, it could represent a significant advancement in the diagnostic landscape for ALL. Additionally, the question of whether these lncRNAs can be detected at earlier time points in patients raises the possibility of their utility in early diagnosis, potentially enabling timely interventions and improved patient outcomes. This dual exploration not only delves into the collaborative potential of these lncRNAs with other biomarkers but also considers their temporal detectability, offering insights into the broader clinical applicability and diagnostic advancements they may bring to the field of ALL diagnosis.

One of the major limitations of our study is a somewhat low number of candidate lncRNAs. According to Deng et al., the expression of MYC can affect the expression of target/downstream lncRNAs.^56^ There are plenty of candidates that need to be investigated in this regard. Secondly, there is a snippet of information regarding the roles of GJA9‐MYCBP in cellular and molecular processes, hence, we believe that some upcoming investigations can address this limitation. Last but not least, a small number of patients had been recruited for this study that in most cases, a long time follow‐up was not possible, therefore, further studies are needed to show whether the candidate lncRNAs may be used as a prognostic biomarker in ALL.

## 5 CONCLUSIONS

In essence, in this study, we showed that the expression levels of MYC, lncRNA GJA9‐MYCBP, and PVT1 were increased in ALL patients compared to the control individuals, however, the exact functions of each lncRNA and the molecular mechanisms whereby these lncRNA function during ALL development are still not clearly understood. We also proposed that these lncRNAs may be used as ALL diagnostic biomarkers. However, large‐scale studies are required to verify the clinical and molecular significance of these potential biomarkers in ALL and maybe in other leukemias.

### 5.1 Suggestion

The upregulation of lncRNA GJA9‐MYCBP and PVT1, along with increased MYC mRNA levels in ALL samples compared to healthy controls, suggests their potential role as diagnostic biomarkers for ALL. The satisfactory diagnostic efficacy indicated by the ROC curve analysis supports this hypothesis. Furthermore, the positive correlations revealed by linear regression analysis between MYC expression and lncRNA GJA9‐MYCBP and PVT1 in ALL patients strengthen the association between these molecular factors in the context of ALL. Considering the findings, further research could explore the functional implications of lncRNA GJA9‐MYCBP and PVT1 in ALL pathogenesis. Investigating potential therapeutic interventions targeting these molecules may also be valuable for developing targeted treatments for ALL patients with elevated MYC expression and upregulated lncRNAs.

## AUTHOR CONTRIBUTIONS

**Kamal Shahamiri:** Conceptualization (equal); data curation (equal); formal analysis (equal); funding acquisition (equal); investigation (equal). **Arash Alghasi:** Conceptualization (supporting); methodology (lead); supervision (lead); validation (lead). **Najmaldin Saki:** Methodology (equal); project administration (equal); supervision (equal); validation (equal). **Hossein Teimori:** Conceptualization (equal); project administration (equal); supervision (equal); visualization (equal); writing – review and editing (equal). **Gholam Abbas Kaydani:** Investigation (equal); methodology (equal); project administration (equal); validation (equal); visualization (equal). **Setare sheikhi:** Investigation (equal); visualization (equal); writing – original draft (equal).

## FUNDING INFORMATION

This study was financially granted by the Research Fund of Shahrekord University of Medical Science (5677).

## CONFLICT OF INTEREST STATEMENT

The authors declare that they have no competing interests.

## ETHICS STATEMENT

All procedures performed in studies involving human participants were in accordance with the ethical standards of the institutional and/or national research committee of Shahrekord University of Medical Science (IR.SKUMS.REC.1399.273). This article does not contain any studies with animals performed by any of the authors.

## INFORMED CONSENT

Informed consent was obtained from all individual participants included in the study.

## Supporting information


**Data S1.** Supporting Information.

## Data Availability

Data sharing is not applicable to this article as no new data were created or analyzed in this study.

## References

[1] Dorantes‐Acosta E , Pelayo R . Lineage switching in acute leukemias: a consequence of stem cell plasticity? Bone Marrow Res. 2012;2012:1‐18. 10.1155/2012/406796 PMC3407598 22852088

[2] Mirali S , Schimmer AD . The role of mitochondrial proteases in leukemic cells and leukemic stem cells. Stem Cells Transl Med. 2020;9(12):1481‐1487. 32761807 10.1002/sctm.20-0142 PMC7695628

[3] Brillantino C , Rossi E , Bifano D , et al. An unusual onset of pediatric acute lymphoblastic leukemia. J Ultrasound. 2021;24(4):555‐560. 32328840 10.1007/s40477-020-00461-y PMC8572300

[4] Quan X , Zhang N , Chen Y , Zeng H , Deng J . Development of an immune‐related prognostic model for pediatric acute lymphoblastic leukemia patients. Mol Genet Genomic Med. 2020;8(9):e1404. 32666718 10.1002/mgg3.1404 PMC7507390

[5] Topp MS , Gökbuget N , Zugmaier G , et al. Long‐term survival of patients with relapsed/refractory acute lymphoblastic leukemia treated with blinatumomab. Cancer. 2021;127(4):554‐559. 33141929 10.1002/cncr.33298 PMC7894150

[6] Bárcenas‐López DA , Núñez‐Enríquez JC , Hidalgo‐Miranda A , et al. Transcriptome analysis identifies LINC00152 as a biomarker of early relapse and mortality in acute lymphoblastic leukemia. Genes. 2020;11(3):302. 32183133 10.3390/genes11030302 PMC7140896

[7] Bitaraf A , Razmara E , Bakhshinejad B , et al. The oncogenic and tumor suppressive roles of RNA‐binding proteins in human cancers. J Cell Physiol. 2021;236:6200‐6224. 33559213 10.1002/jcp.30311

[8] Mahgoub EO , Razmara E , Bitaraf A , et al. Advances of exosome isolation techniques in lung cancer. Mol Biol Rep. 2020;47:7229‐7251. 32789576 10.1007/s11033-020-05715-w

[9] Poursheikhani A , Bahmanpour Z , Razmara E , et al. Non‐coding RNAs underlying chemoresistance in gastric cancer. Cell Oncol. 2020;43:961‐988. 10.1007/s13402-020-00528-2 PMC12990740 32495294

[10] Tahmouresi F , Razmara E , Pakravan K , et al. Upregulation of the long noncoding RNAs DSCAM‐AS1 and MANCR is a potential diagnostic marker for breast carcinoma. Biotechnol Appl Biochem. 2020;68(6):1250‐1256. 10.1002/bab.2048 33012018

[11] Razmara E , Bitaraf A , Karimi B , Babashah S . Functions of the SNAI family in chondrocyte‐to‐osteocyte development. Ann N Y Acad Sci. 2021;1503:5‐22. 34403146 10.1111/nyas.14668

[12] Razmara E , Bitaraf A , Yousefi H , et al. Non‐coding RNAs in cartilage development: an updated review. Int J Mol Sci. 2019;20(18):4475. 31514268 10.3390/ijms20184475 PMC6769748

[13] Razmara E , Salehi M , Aslani S , et al. Graves' disease: introducing new genetic and epigenetic contributors. J Mol Endocrinol. 2021;66(2):R33‐R55. 33295879 10.1530/JME-20-0078

[14] Hao Q , Zong X , Sun Q , et al. The S‐phase‐induced lncRNA SUNO1 promotes cell proliferation by controlling YAP1/Hippo signaling pathway. Elife. 2020;9:e55102. 33108271 10.7554/eLife.55102 PMC7591261

[15] Gao J , Wang F , Wu P , Chen Y , Jia Y . Aberrant LncRNA expression in leukemia. J Cancer. 2020;11(14):4284‐4296. 32368311 10.7150/jca.42093 PMC7196264

[16] Delgado MD , León J . Myc roles in hematopoiesis and leukemia. Genes Cancer. 2010;1(6):605‐616. 21779460 10.1177/1947601910377495 PMC3092227

[17] Lee DH , Kwon NE , Lee WJ , et al. Increased O‐GlcNAcylation of c‐Myc promotes pre‐B cell proliferation. Cells. 2020;9(1):158. 31936366 10.3390/cells9010158 PMC7016991

[18] Iaccarino I . lncRNAs and MYC: an intricate relationship. Int J Mol Sci. 2017;18(7):1497. 28704924 10.3390/ijms18071497 PMC5535987

[19] Chen Q , Shen H , Zhu X , et al. A nuclear lncRNA Linc00839 as a Myc target to promote breast cancer chemoresistance via PI3K/AKT signaling pathway. Cancer Sci. 2020;111(9):3279‐3291. 32619088 10.1111/cas.14555 PMC7469761

[20] Shigeyasu K , Toden S , Ozawa T , et al. The PVT1 lncRNA is a novel epigenetic enhancer of MYC, and a promising risk‐stratification biomarker in colorectal cancer. Mol Cancer. 2020;19(1):1‐6. 33148262 10.1186/s12943-020-01277-4 PMC7643275

[21] Qian J , Garg A , Li F , Shen Q , Xiao K . lncRNA LUNAR1 accelerates colorectal cancer progression by targeting the miR‐495‐3p/MYCBP axis. Int J Oncol. 2020;57(5):1157‐1168. 33300052 10.3892/ijo.2020.5128 PMC7549538

[22] Wei Y , Wang Z , Zong Y , Deng D , Chen P , Lu J . LncRNA MFI2‐AS1 promotes HCC progression and metastasis by acting as a competing endogenous RNA of miR‐134 to upregulate FOXM1 expression. Biomed Pharmacother. 2020;125:109890. 32106369 10.1016/j.biopha.2020.109890

[23] Sun H , Zhang Z , Luo W , Liu J , Lou Y , Xia S . MiR‐7 functions as a tumor suppressor by targeting the oncogenes TAL1 in T‐cell acute lymphoblastic leukemia. Technol Cancer Res Treat. 2020;19:1533033820934130. 32633635 10.1177/1533033820934130 PMC7343363

[24] Tseng Y‐Y , Bagchi A . The PVT1‐MYC duet in cancer. Mol Cell Oncol. 2015;2(2):e974467. 27308428 10.4161/23723556.2014.974467 PMC4904896

[25] Zhou Q , Chen F , Zhao J , et al. Long non‐coding RNA PVT1 promotes osteosarcoma development by acting as a molecular sponge to regulate miR‐195. Oncotarget. 2016;7(50):82620‐82633. 27813492 10.18632/oncotarget.13012 PMC5347719

[26] Ding J , Li D , Gong M , et al. Expression and clinical significance of the long non‐coding RNA PVT1 in human gastric cancer. Onco Targets Ther. 2014;7:1625. 25258543 10.2147/OTT.S68854 PMC4172193

[27] Yang Y‐R , Zang SZ , Zhong CL , Li YX , Zhao SS , Feng XJ . Increased expression of the lncRNA PVT1 promotes tumorigenesis in non‐small cell lung cancer. Int J Clin Exp Pathol. 2014;7(10):6929‐6935. 25400777 PMC4230094

[28] Iden M , Fye S , Li K , Chowdhury T , Ramchandran R , Rader JS . The lncRNA PVT1 contributes to the cervical cancer phenotype and associates with poor patient prognosis. PLoS One. 2016;11(5):e0156274. 27232880 10.1371/journal.pone.0156274 PMC4883781

[29] Chen X , Gao G , Liu S , et al. Long noncoding RNA PVT1 as a novel diagnostic biomarker and therapeutic target for melanoma. Biomed Res Int. 2017;2017:7038579. 10.1155/2017/7038579 PMC5318621 28265576

[30] Fan H , Zhu JH , Yao XQ . Knockdown of long non‐coding RNA PVT1 reverses multidrug resistance in colorectal cancer cells. Mol Med Rep. 2018;17(6):8309‐8315. 29693171 10.3892/mmr.2018.8907 PMC5984006

[31] Hu J , Han Q , Gu Y , et al. Circular RNA PVT1 expression and its roles in acute lymphoblastic leukemia. Epigenomics. 2018;10(6):723‐732. 29693417 10.2217/epi-2017-0142

[32] Jin K , Wang S , Zhang Y , et al. Long non‐coding RNA PVT1 interacts with MYC and its downstream molecules to synergistically promote tumorigenesis. Cell Mol Life Sci. 2019;76(21):4275‐4289. 31309249 10.1007/s00018-019-03222-1 PMC6803569

[33] Arber DA , Orazi A , Hasserjian R , et al. The 2016 revision to the World Health Organization classification of myeloid neoplasms and acute leukemia. Blood. 2016;127(20):2391‐2405. 27069254 10.1182/blood-2016-03-643544

[34] Lejhancova‐Tousovska K , Zapletal O , Vytiskova S , Strbackova P , Sterba J . Profile of thrombin generation in children with acute lymphoblastic leukemia treated by Berlin–Frankfurt–Münster (BFM) protocols. Blood Coagul Fibrinolysis. 2012;23(2):144‐154. 22227959 10.1097/MBC.0b013e32834fb539

[35] Cheng L , Wang P , Tian R , et al. LncRNA2Target v2. 0: a comprehensive database for target genes of lncRNAs in human and mouse. Nucleic Acids Res. 2019;47(D1):D140‐D144. 30380072 10.1093/nar/gky1051 PMC6323902

[36] Chen J , Zhang J , Gao Y , et al. LncSEA: a platform for long non‐coding RNA related sets and enrichment analysis. Nucleic Acids Res. 2021;49(D1):D969‐D980. 33045741 10.1093/nar/gkaa806 PMC7778898

[37] Paraskevopoulou MD , Vlachos IS , Karagkouni D , et al. DIANA‐LncBase v2: indexing microRNA targets on non‐coding transcripts. Nucleic Acids Res. 2016;44(D1):D231‐D238. 26612864 10.1093/nar/gkv1270 PMC4702897

[38] Zhang W , Yue X , Tang G , Wu W , Huang F , Zhang X . SFPEL‐LPI: sequence‐based feature projection ensemble learning for predicting LncRNA‐protein interactions. PLoS Comput Biol. 2018;14(12):e1006616. 30533006 10.1371/journal.pcbi.1006616 PMC6331124

[39] Lin Y , Liu T , Cui T , et al. RNAInter in 2020: RNA interactome repository with increased coverage and annotation. Nucleic Acids Res. 2020;48(D1):D189‐D197. 31906603 10.1093/nar/gkz804 PMC6943043

[40] Livak KJ , Schmittgen TD . Analysis of relative gene expression data using real‐time quantitative PCR and the 2− ΔΔCT method. Methods. 2001;25(4):402‐408. 11846609 10.1006/meth.2001.1262

[41] Nagler A , Baron F , Labopin M , et al. Measurable residual disease (MRD) testing for acute leukemia in EBMT transplant centers: a survey on behalf of the ALWP of the EBMT. Bone Marrow Transplant. 2021;56(1):218‐224. 32724200 10.1038/s41409-020-01005-y

[42] Dong Y , Shi O , Zeng Q , et al. Leukemia incidence trends at the global, regional, and national level between 1990 and 2017. Exp Hematol Oncol. 2020;9(1):1‐11. 32577323 10.1186/s40164-020-00170-6 PMC7304189

[43] Hong Z , Wei Z , Xie T , et al. Targeting chemokines for acute lymphoblastic leukemia therapy. J Hematol Oncol. 2021;14(1):1‐14. 33743810 10.1186/s13045-021-01060-y PMC7981899

[44] Li G , Gao L , Zhao J , Liu D , Li H , Hu M . LncRNA ANRIL/miR‐7‐5p/TCF4 axis contributes to the progression of T cell acute lymphoblastic leukemia. Cancer Cell Int. 2020;20(1):1‐12. 32714094 10.1186/s12935-020-01376-8 PMC7376839

[45] Xiao S , Xu N , Ding Q , Huang S , Zha Y , Zhu H . LncRNA VPS9D1‐AS1 promotes cell proliferation in acute lymphoblastic leukemia through modulating GPX1 expression by miR‐491‐5p and miR‐214‐3p evasion. Biosci Rep. 2020;40(10):BSR20193461. 32808668 10.1042/BSR20193461 PMC7536331

[46] Reizes O , Alban T , Horowitz M , et al. Activation of Adaptive Immune Cells Is an Early Response to Hyperthermic Intraperitoneal Chemotherapy Treatment in Ovarian Cancer Research Square. Preprint, 04 August 2021. doi:10.21203/rs.3.rs-701286/v1

[47] Zhou C , Yi C , Yi Y , et al. LncRNA PVT1 promotes gemcitabine resistance of pancreatic cancer via activating Wnt/β‐catenin and autophagy pathway through modulating the miR‐619‐5p/Pygo2 and miR‐619‐5p/ATG14 axes. Mol Cancer. 2020;19(1):1‐24. 32727463 10.1186/s12943-020-01237-y PMC7389684

[48] Cui M , You L , Ren X , Zhao W , Liao Q , Zhao Y . Long non‐coding RNA PVT1 and cancer. Biochem Biophys Res Commun. 2016;471(1):10‐14. 26850852 10.1016/j.bbrc.2015.12.101

[49] Wang A , Zhang T , Wei W , et al. The long noncoding RNA LINC00665 facilitates c‐Myc transcriptional activity via the miR‐195‐5p MYCBP Axis to promote progression of lung adenocarcinoma. Front Oncol. 2021;11:2530. 10.3389/fonc.2021.666551 PMC8281894 34277412

[50] Li C , Tan F , Pei Q , et al. Non‐coding RNA MFI2‐AS1 promotes colorectal cancer cell proliferation, migration and invasion through miR‐574‐5p/MYCBP axis. Cell Prolif. 2019;52(4):e12632. 31094023 10.1111/cpr.12632 PMC6668983

[51] Gong L , Xia Y , Qian Z , et al. Overexpression of MYC binding protein promotes invasion and migration in gastric cancer. Oncol Lett. 2018;15(4):5243‐5249. 29552163 10.3892/ol.2018.7944 PMC5840499

[52] Ikoma D , Cardillo N , Devor E , Gonzalez‑bosquet J . A nuclear polymorphism at the 8q24 region is associated with improved survival time and chemo‐response in high‐grade serous ovarian cancer. Oncol Lett. 2021;22(4):1‐9. 10.3892/ol.2021.12994 PMC8371958 34429773

[53] Martínez‐Barriocanal Á , Arango D , Dopeso H . PVT1 long non‐coding RNA in gastrointestinal cancer. Front Oncol. 2020;10:38. 32083000 10.3389/fonc.2020.00038 PMC7005105

[54] Houshmand M , Yazdi N , Kazemi A , et al. Long non‐coding RNA PVT1 as a novel candidate for targeted therapy in hematologic malignancies. Int J Biochem Cell Biol. 2018;98:54‐64. 29510227 10.1016/j.biocel.2018.03.001

[55] Takahashi Y , Sawada G , Kurashige J , et al. Amplification of PVT‐1 is involved in poor prognosis via apoptosis inhibition in colorectal cancers. Br J Cancer. 2014;110(1):164‐171. 24196785 10.1038/bjc.2013.698 PMC3887297

[56] Deng K , Guo X , Wang H , Xia J . The lncRNA‐MYC regulatory network in cancer. Tumour Biol. 2014;35(10):9497‐9503. 25139102 10.1007/s13277-014-2511-y

