# Supplementary material for: Upregulation of the long noncoding RNA GJA9‐MYCBP and PVT1 is a potential diagnostic biomarker for acute lymphoblastic leukemia
Source: Cancer Rep (Hoboken). 2024 Jul 12;7(7):e2115. doi: 10.1002/cnr2.2115 (PMC11240143; doi:10.1002/cnr2.2115)
Supplement: Supplementary file 1 — Data S1. Supporting Information. [file CNR2-7-e2115-s001.docx]

| **Age (years)** | | | | **Gender** | | **Groups** |
| --- | --- | --- | --- | --- | --- | --- |
| **11-16** | **6-11** | | **1-5** | **Man** | **Female** |  |
| 13 | 11 | 16 | | 26 | 14 |  |
| 8 | 15 | 17 | | 21 | 19 | **The witness** |
| 0.163 | 0.096 | 0.741 | | 0.058 | 0.066 | **P** |

**Supplementary Table.** Different interacting molecules were predicted as the target for the lncRNA GJA9-MYCBP and PVT1. There were 22 patianets B-All and 8 patients with T-All.


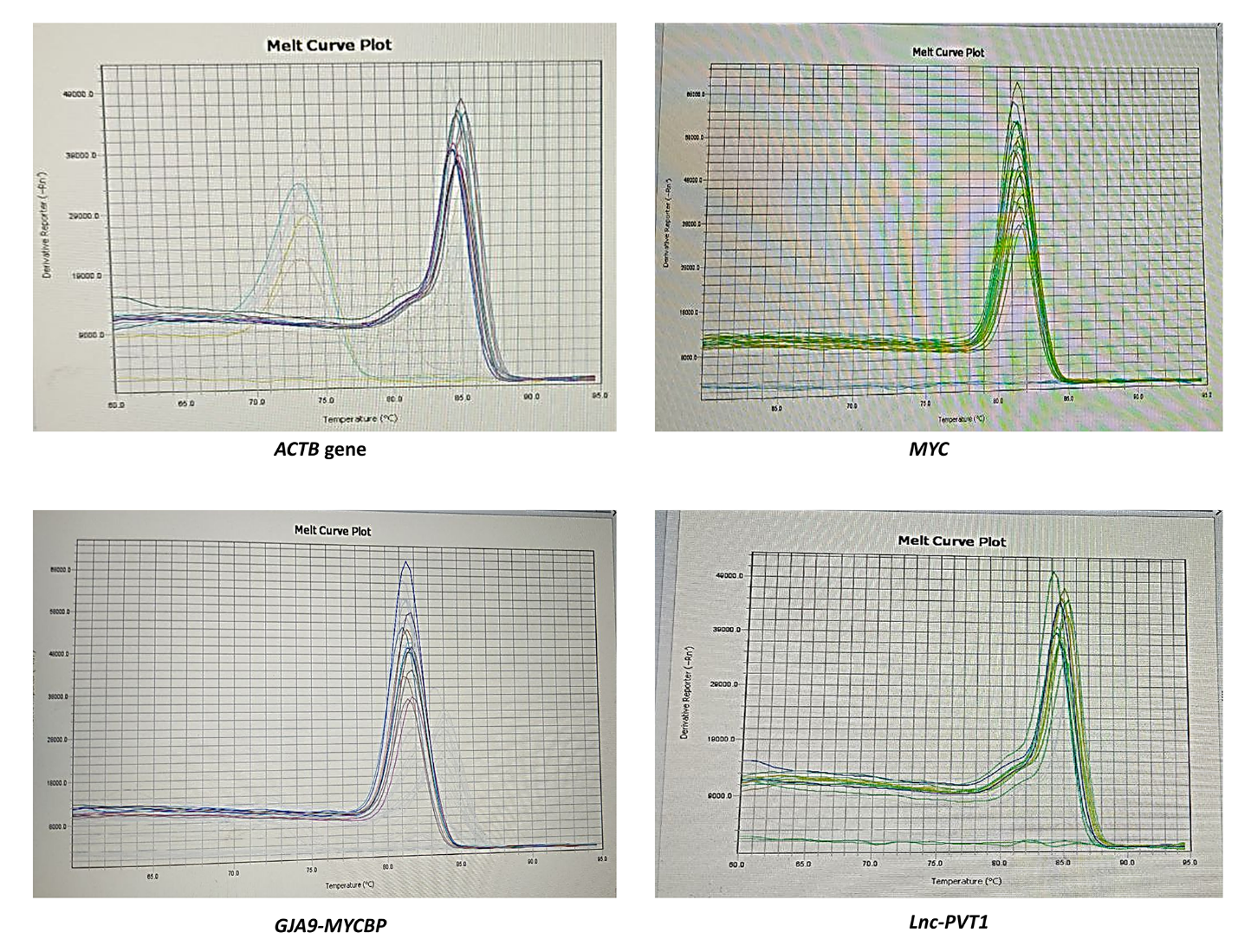


**Supplementary Figure 1.** Melting curves were used to analyze the specificity of PCR products
